# Supplementary material for: Comparing the evolutionary dynamics of predominant SARS-CoV-2 virus lineages co-circulating in Mexico
Source: eLife. 2023 Jul 27;12:e82069. doi: 10.7554/eLife.82069 (PMC10431917; doi:10.7554/eLife.82069)
Supplement: Transparent reporting form [file elife-82069-transrepform1.pdf]

1st Floor
24 Hills Road
Cambridge CB2 1JP, UK

P 01223 855340
W elifesciences.org
T @elife

eLife's transparent reporting form

We encourage authors to provide detailed information *within their submission* to facilitate the interpretation and replication of experiments. Authors can upload supporting documentation to indicate the use of appropriate reporting guidelines for health-related research (see EQUATOR Network), life science research (see the BioSharing Information Resource), or the ARRIVE guidelines for reporting work involving animal research. Where applicable, authors should refer to any relevant reporting standards documents in this form.

If you have any questions, please consult our Journal Policies and/or contact us: editorial@elifesciences.org.

Sample-size estimation

- You should state whether an appropriate sample size was computed when the study was being designed
- You should state the statistical method of sample size computation and any required assumptions
- If no explicit power analysis was used, you should describe how you decided what sample (replicate) size (number) to use

Please outline where this information can be found within the submission (e.g., sections or figure legends), or explain why this information doesn't apply to your submission:

Dataset construction involves a uniform spatio-temporal sequence subsampling that is fully described in Methods Section 2. The pipeline then uses a phylogenetically informed subsampling to reduce the size and redundancy of the inferred trees with a minimal loss of diversity. This migration and phylogenetically-informed sequence sampling approach, coupled with balancing the number of sequences from different locations, aims to mitigate the effects of geographical over-representation impacting phylogeographic analyses, in which clades tend to not segregate fully, and certain regions may appear as more frequent seeding sources than they are in reality. We have ensured to include as many sequences as possible to allow for computationally feasible analyses.

Replicates

- You should report how often each experiment was performed
- You should include a definition of biological versus technical replication
- The data obtained should be provided and sufficient information should be provided to indicate the number of independent biological and/or technical replicates
- If you encountered any outliers, you should describe how these were handled
- Criteria for exclusion/inclusion of data should be clearly stated
- High-throughput sequence data should be uploaded before submission, with a private link for reviewers provided (these are available from both GEO and ArrayExpress)

Please outline where this information can be found within the submission (e.g., sections or figure legends), or explain why this information doesn't apply to your submission:

All information related to data collation and initial sequence subsampling (including the description of the exclusion/inclusion criteria), dataset construction, and further analysis in which outliers need to be assessed (namely time-scaled analysis), are fully described in the Methods Section (1-4). The distribution of genome sequences for the different datasets before and after subsampling under a migration and phylogenetically-informed approach are available in Supplementary Figure 2. Root-to-tip regression plots for the trees displaying the distribution of sequences within datasets relative to genetic diversity and sampling across time are available in Supplementary Figure 3.

eLife Sciences Publications, Ltd is a limited liability non-profit non-stock corporation incorporated in the State of Delaware, USA, with company number 5030732, and is registered in the UK with company number FC030576 and branch number BR015634 at the address 1st Floor, 24 Hills Road, Cambridge CB2 1JP | August 2014

Statistical reporting

- Statistical analysis methods should be described and justified
- Raw data should be presented in figures whenever informative to do so (typically when N per group is less than 10)
- For each experiment, you should identify the statistical tests used, exact values of N, definitions of center, methods of multiple test correction, and dispersion and precision measures (e.g., mean, median, SD, SEM, confidence intervals; and, for the major substantive results, a measure of effect size (e.g., Pearson's r, Cohen's d)
- Report exact p-values wherever possible alongside the summary statistics and 95% confidence intervals. These should be reported for all key questions and not only when the p-value is less than 0.05.

Please outline where this information can be found within the submission (e.g., sections or figure legends), or explain why this information doesn't apply to your submission:

All information related to statistical analysis, including sample sizes (L421-423, 472-484) are fully described in the Methods Section 1-3. The correlation between the cumulative number of cases per state compared to the number of viral genome sequences generated in Mexico up to March 2022 (including values for Spearman's, Pearson's and R2) are reported in Supplementary Figure 1. Statistical testing within our study is inherent of Bayesian Inference (fully described in Methods Section 4). This includes reporting effective sample size values, and Bayes Factor Rate values (Supplementary Table 1). All derived estimates are reported with 95% HPD intervals throughout the results section, whilst statistical limitations of our analytical approach regarding the time-scales analyses are also stated in L495-499.

(For large datasets, or papers with a very large number of statistical tests, you may upload a single table file with tests, Ns, etc., with reference to sections in the manuscript.)

Group allocation

- Indicate how samples were allocated into experimental groups (in the case of clinical studies, please specify allocation to treatment method); if randomization was used, please also state if restricted randomization was applied
- Indicate if masking was used during group allocation, data collection and/or data analysis

Please outline where this information can be found within the submission (e.g., sections or figure legends), or explain why this information doesn't apply to your submission:

NA

Additional data files ("source data")

- We encourage you to upload relevant additional data files, such as numerical data that are represented as a graph in a figure, or as a summary table
- Where provided, these should be in the most useful format, and they can be uploaded as "Source data" files linked to a main figure or table
- Include model definition files including the full list of parameters used
- Include code used for data analysis (e.g., R, MatLab)
- Avoid stating that data files are "available upon request"

Please indicate the figures or tables for which source data files have been provided:

Source data: virus genome IDs and GISAID accession numbers for the sequences used within each dataset are provided in the Supplementary Data 1 file. All genomic and epidemiological data supporting the findings of this study is publicly available. Our bioinformatic pipeline implementing a migration data and phylogenetically-informed sequence subsampling approach is publicly available at https://github.com/rhysinward/Mexico_subsampling. All source data is available as supplementary data or in the Supplementary Information File.
